# Class B scavenger receptor resists WSSV replication by recognizing the viral lipid molecule and promoting phagocytosis

**DOI:** 10.1128/jvi.01700-24

**Published:** 2025-02-05

**Authors:** Yi-Heng Huang, Xin-Lu Guo, Meng-Ke Shan, Gui-Wen Yang, Hui-Ting Yang

**Affiliations:** 1 Shandong Provincial Key Laboratory of Animal Resistance Biology, College of Life Sciences, Shandong Normal University 47856 , Jinan, China; The University of Arizona, Tucson, Arizona, USA

**Keywords:** *Pc*SRB, WSSV, recognition, phagocytosis

## Abstract

Class B scavenger receptors (SRBs) have been well-studied in bacteria-induced immune responses in invertebrates. However, the status of SRB-defending viruses remains unclear. In this study, we identified a scavenger receptor in *Procambarus clarkii* (crayfish), which is homologous to mammalian SRBs, and designated it as *Pc*SRB. The expression of *Pc*SRB was upregulated after the WSSV challenge. The survival rate of crayfish was decreased, but the WSSV copy number increased after *Pc*SRB knockdown during virus invasion. In addition, *Pc*SRB bound to WSSV. Furthermore, we detected how *Pc*SRB interacted with WSSV, and we found that *Pc*SRB could bind to cholesta-3,5-diene, (CD3,5), a novel WSSV lipid ligand, rather than dibutyl phthalate (DBP). Besides, *Pc*SRB could bind to VP19, VP26, and VP28, rather than VP24. Mutant-binding experiments demonstrated that the hydrophobic domain (130–180 aa) of *Pc*SRB is important for recognizing WSSV. Furthermore, *Pc*SRB might promote lysosomal eliminating function to degrade WSSV. Altogether, we identified a new mechanism for scavenger receptor recognition and resistance to WSSV.

**IMPORTANCE:**

*Pc*SRB could bind to WSSV directly. *Pc*SRB could interact with WSSV via binding to lipid molecule CD3,5 and viral envelope proteins. *Pc*SRB could influence lysosomal activation.

## INTRODUCTION

Class B scavenger receptors (SRBs) are transmembrane receptors. Structurally, SRBs consist of two transmembrane domains, two very short cytoplasmic domains, and a large glycosylated extracellular domain (1). The extracellular domain is considered the interaction region for binding to ligands. The reported SRBs are classified as SRB1, SRB2 (CD36), and SRB3 (SCARB2) (1, 2). Certain SRBs from different species have been reported to be involved in lipoprotein transportation and pathogen recognition (3, 4).

SRBs are important for virus-induced innate immunity. SRB1 can recognize the hepatitis C virus (HCV) envelope glycoprotein E2 (5, 6). The *SR-B1* mutation (S112F) decreases the infectivity of HCV derived from HEK 293T cells expressing the E2 protein (7). High-density lipoprotein (HDL) components bind to SRB1 located on the cell surface. The binding of HDL to SRB1 promotes the entry of SARS-CoV-2 into several cell lines (4). Moreover, SRB2 mediates HDL endocytosis via a clathrin-dependent pathway (8). SRB1 facilitates dengue virus evasion into cells via apolipoprotein A-I mediation (9). SRB3 is the receptor of several enteroviral species, such as coxsackievirus and enterovirus 71 (8, 10, 11). The expression of SRB2 is upregulated in C33A cells stably expressing the HPV16 E7 protein (12). The SRB1 demonstrated binding ability to the virus in turbot (*Scophthalmus maximus* L.). However, it remains unclear which viral molecules bind to SRB1 (13). Previous reports have demonstrated that SRBs are involved in different types of viral invasion. Lipoproteins could be possible cooperators with SRBs in virus recognition.

WSSV is a destructive crustacean pathogen. Several studies have attempted to elucidate this recognition mechanism. Most previous studies have reported that the envelope and nucleocapsid proteins are important for the host recognition of viruses. The cell laminin receptor (Lamr) interacts with the WSSV structural protein VP31 in black tiger shrimp (*Penaeus monodon*) (14). The C-Type Lectin FmLC6 in banana shrimp (*Fenneropenaeus merguiensis*) can directly bind to VP15, VP39A, and VP28 with different affinities (15). B-integrin has been reported to interact with the WSSV envelope proteins VP26, VP31, VP37, and VP90 and nucleocapsid protein VP136 in white-leg shrimp (16). The scavenger receptor C (*Mj*SRC) obtained from kuruma shrimp interacts with the viral envelope protein (VP19) and initiates antiviral responses in shrimp (17). Vago from prawns (*Macrobrachium nipponense*) was reported as a pattern recognition receptor that recognizes VP26 and VP28 and activates the JAK/STAT pathway (18). Toll4 from white shrimp (*Litopenaeus vannamei*) is known to restrict WSSV infection (19). Chondroitin proteoglycan 2 interacts with VP26 and VP28 to facilitate WSSV adhesion (20). According to previous reports, host receptors largely recognize WSSV by interacting with its structural proteins.

Lipids are present in the envelope of viruses. Envelope lipids are derived from the host membrane structures (21). WSSV lipid components were analyzed after virus discovery. The viral lipid extract comprises approximately 22% of the dry weight of the virus. Phosphatidylcholine and phosphatidylethanolamine comprise 62.9% and 25.8% of the WSSV phospholipids, respectively. They comprise 58.5% and 30% of the crayfish nuclei phospholipids, respectively. In total, 15 neutral lipids are detected in the WSSV. However, only 11 neutral lipids have been detected in crayfish membrane structures (22). Therefore, the four types of neutral lipids are different from host lipids. However, it remains unclear whether different lipids are involved in viral recognition.

In this study, we identified scavenger receptor B (*Pc*SRB) in *Procambrus clarkii*. The *Pc*SRB restricted WSSV replication to protect the host. We found that *Pc*SRB recognized WSSV by interacting with viral envelope lipid molecules and proteins. Furthermore, mutant *Pc*SRB demonstrated that the hydrophobic domain of *Pc*SRB was vital for recognizing WSSV. In addition, *Pc*SRB promoted the inhibition of WSSV replication by lysosomes. This study revealed a novel pattern for the elimination of WSSV.

## MATERIALS AND METHODS

### Animals and pathogen challenge

The crayfish (10–15 g) used in the study was purchased from the Jinan Seafood Market in Shandong province. The crayfish was maintained in the aquarium with freshwater at 22°C–27°C. Intact WSSV particles were acquired as described in a previous study (23). Each crayfish was injected with 1 × 10^5^ WSSV virions during the challenge experiments (17).

### Tissue collection and cDNA synthesis

Six tissues (hemocytes, heart, hepatopancreas, gills, stomach, and intestine) were collected at different time points (0, 6, 12, 24, and 48 h) after the WSSV challenge. All crayfish tissues were homogenized in homogenizers, and total RNA was extracted using the TRIzol reagent (TIANGEN, China, DP424). Total RNA extraction and cDNA synthesis were performed as described in a previous study (24).

### Quantitative real-time PCR (qRT-PCR) analysis

The transcript levels of *PcSRB* were evaluated by quantitative real-time-polymerase chain reaction (qRT-PCR) using the SRB-RT-F and SRB-RT-R primers (Table S1). The 18S ribosome RNA was the internal control gene. The qRT-PCR procedure was performed following the instruction of SYBR Green Pro Taq HS (AG, China, AG11701) in a qRT-PCR instrument (LightCycler96, Roche, Switzerland). The PCR procedure was as follows: 95°C for 10 min; 40 cycles of 94°C for 15 s, and 60°C for 60 s; and a melting curve analysis from 72 °C to 95°C. Each test was performed in triplicate. Data were calculated using the 2^-∆∆Ct^ method for three independent experiments. Additionally, *t*-test was used for significance testing (* 0.01 < *P* < 0.05, ** *P* < 0.01).

### Sequence analysis of *Pc*SRB

The full-length *Pc*SRB sequence was obtained using transcriptome analysis (MajorBio, Shanghai, China). The amino acid sequence of *Pc*SRB was predicted using ExPASy-Translate (https://web.expasy.org/translate/). The structural and functional domain characteristics of *Pc*SRB were analyzed using SMART (http://smart.embl.de/). The molecular weight and isoelectric point of *Pc*SRB were predicted using DNASTAR software. The phylogenetic trees of *Pc*SRB and the scavenger receptors (SRs) of other species were analyzed using MEGA6.0 software (25). The three-dimensional model of *Pc*SRB protein was constructed using SWISS-MODEL (https://swissmodel.expasy.org/).

### Expression and purification of recombinant *Pc*SRB

The total cDNA was used as the template. The primers, SRB-BamHI-F and SRB-XhoI-R, were used for *Pc*SRB gene amplification. The PCR program was: 95°C for 1 min, 95°C for 20 s, 55°C for 20 s, 70°C for 60 s, 38 cycles; 70°C for 10 min. The resulting PCR product and pET-32a(+) plasmid were digested with BamHI and XhoI. The digested product was cloned into the pET-32a(+) plasmid. The *Pc*SRB protein was expressed and purified as described in a previous study (26).

### Detection of WSSV copy number

The number of WSSV copies in tissues was determined as described previously (27). The viral *VP*28 DNA was inserted into the pLB plasmid (TIANGEN, Beijing, China, VT205). The original plasmid concentration was quantified using a spectrophotometer (NanoDrop 2000c, Thermo, USA) and subsequently diluted to 10^1^, 10^2^, 10^3^, 10^4^, 10^5^¸ and 10^6^ copies/μL. Q-PCR was performed to determine cycle thresholds (CT). A standard curve was established based on the plasmid copy number and CT results. The absolute copy number of WSSV in the tissues was calculated using a standard curve.

### RNA interference and survival rate assay

To decrease the expression of *Pc*SRB, RNA interference experiments were performed as previously described (24). Ds*PcSRB* DNA was synthesized using SRBi-F and SRBi-R primers (Table S1). The dsRNA was synthesized using a T7 transcription kit (Thermo Fisher Scientific, USA, K0441). Purified dsRNA (30 µg) was injected into the abdomen of the crayfish. The dsRNA of the green fluorescent protein gene (dsGFP) was also injected into crayfish, which constituted the mock group. *PcSRB* mRNA levels were detected using qRT-PCR at 24 h after dsRNA injection. WSSV was injected into the crayfish at 24 h after dsRNA injection. The gills were collected for *VP28* expression analysis and copy number determination at 24 h after WSSV challenge. Three crayfish were used for WSSV copy number determination. Dead crayfish were counted at different time points (1, 2, 3, 4, and 5 days). Thirty crayfish were used for the survival rate assay.

### Transmission electron microscope (TEM) assay

Colloidal gold-labeled proteins were prepared as described previously (17). The recombinant *Pc*SRB protein was incubated with colloidal gold (donated by Professor Lu) and PEG for 30 min. The labeled protein was washed three times using phosphate buffered solution (PBS, containing 1% PEG). Purified WSSV was incubated on a carbon film-coated copper mesh for 15 min. Colloidal gold-labeled proteins (1 mg/mL) were incubated with a copper mesh for 30 min at 25°C. To remove the saline ions, the sample was washed five times with double-distilled water. The samples were incubated using uranyl acetate (SPI, USA, 6159440) for 30 s and dried in CO_2_. The samples were observed using a TEM (HT-7800, Hitachi, Japan).

### Cell phagocytosis assay

HeLa cells were cultured in the DMEM medium using a conventional method. The target gene *PcSRB* was inserted into the pEGFP-N1 plasmid. The recombinant plasmids were transfected into HeLa cells. DiI (Beyotime, China, C1036)-labeled WSSV was added to the culture medium and incubated for 2 h. Next, DAPI was added and incubated with cells for 15 min. Phagocytosis was detected using a confocal microscope (TCS SP8, Leica, Germany). Hela cells were lysed with RIPA lysis buffer (Beyotime, China, P0013B). The recombinant protein was detected by western blot analysis as in previous study (28). A GFP recombinant rabbit monoclonal antibody (1:5,000, HUABIO, China, ET1607-31) and β-actin monoclonal antibody (1:5,000, ABways, China, AB0035) were the primary antibodies. A HRP-conjugated goat anti-rabbit IgG (1:10,000, Proteintech, China, SA00001) was used as the secondary antibody. The phagocytic rate and index were calculated as follows: Phagocytosis rate = (number of hemocytes containing WSSV in the field of view/number of all hemocytes in the field of view) × 100% and phagocytic index = (number of all WSSV in hemocytes in the field of view/number of all hemocytes in the field of view) × 100%. The phagocytized WSSV and cells were counted in three fields of view.

### Pull-down assay

His-tagged and GST-tagged *Pc*SRB and VPs (VP19, VP24, VP26, and VP28) were expressed as described above. The recombinant proteins were incubated with His-binding resin or glutathione resin (GenScript, Jiangsu, China, L00223I, L00206). Potentially interacting proteins were added to the mixture and incubated for 2 h. The compounds were centrifuged, and the pellet was washed thrice with TBS. The protein complexes were separated by sodium dodecyl sulfate–polyacrylamide gel electrophoresis. Coomassie brilliant blue was used for coloration.

### UV–visible and fluorescence spectrum assay

To preliminary detect the interaction of *Pc*SRB protein and lipid molecules (cholesta-3,5-diene, CD3,5, and dibutyl phthalate, DBP), a UV–absorption spectrum assay was performed. The concentration of recombinant *Pc*SRB (r*Pc*SRB)/His-Tag was 1 µM. The concentration gradient of CD3,5/DBP (Sigma, USA, C6012, 524980) was 0, 20, 40, 60, and 80 µM. His-Tag was used as the mock. In addition, a UV–visible spectral assay was performed. The parameters used in this assay were as follows: scanning range of the emission wavelength 190–500 nm, slit width, 2 nm; and temperature, 300 K. The samples were analyzed using a UV–vis spectrophotometer (TU-1810PC, PERSEE, China). Fluorescence spectra were scanned using a fluorescence spectrometer (Cary Eclipse, Varian, USA). The parameters were as follows: excitation wavelength, 280 nm; wavelength 250–450 nm, excitation and emission slit width, 5 nm; and scan rate, 1,200 nm/min. The concentration rates of CD3,5 and r*Pc*SRB were as follows: 0, 2, 4, 6, 8, and 10.

### Enzyme-linked immunosorbent assay (ELISA)

The interaction of *Pc*SRB protein with lipid molecules CD3,5 and DBP was further detected using ELISA. The 96-well plates were coated with CD3,5 and DBP as previously described (29). A 3% bovine serum albumin (BSA) solution prepared in PBS was incubated with the coated plates at 25°C for 2 h. The r*Pc*SRB protein (0, 5, 15, 30, 50, 80, and 100 nM) was incubated with coated plates for 3 h. The samples were washed using PBS five times. A mouse anti-His-Tag monoclonal antibody (1:5,000, Proteintech, China, 66005–1) was used as the primary antibody. Alkaline phosphatase-conjugated goat anti-mouse IgG (1:10,000, Proteintech, China, SA00002-1) was used as the secondary antibody. The *p*-nitro-phenyl phosphate (10 mM diethanolamine, 0.5 mM MgCl_2_) was added to the wells. The absorbance was measured at 405 nm using a multifunctional microplate reader (FilterMax F3, Molecular Devices Corporation, USA). Purified His-Tag protein was used as a mock control. Each experiment was performed in triplicate.

### Microscale thermophoresis (MST) assay

The MST assay was performed to determine the interaction between lipid molecules and *Pc*SRB. Recombinant *Pc*SRB was labeled using the MO series RED-tris-NTA protein labeling kit (Nona Temper, Germany, MO-L018) according to the manufacturer’s instructions. *Pc*SRB was diluted to 200 nM in PBS-T. A total of 90 µL of the diluted protein solution and 90 µL of dye were incubated for 30 min in the dark. The sample was centrifuged at 15,000 × *g* for 10 min at 4°C, and the supernatant was transferred to a new EP tube. Ligands, CD3,5 and DBP, were diluted in PBS-T. The ligands were diluted in half step-by-step. The labeled proteins were afterward incubated with diluted ligands. A capillary (Nona Temper, Germany, MO-K025) was used to suck the sample onto an MST machine (Monolith NT. 115, NanoTemper, Germany).

### Binding model prediction

The potential binding positions of *Pc*SRB and CD3,5 were identified using AutoDock (4.2.6) (30). The visual structure and exhibition were demonstrated using PyMOL2 software. Hydrophobicity was analyzed using online software (https://web.expasy.org/protscale/).

### Expression and purification of recombinant *Pc*SRB hydrophobic domain deletion mutant and interaction analysis

The mutant gene was built using the PCR method using two pairs of primers, SRB-BamHI-△F1, SRB-△R1, SRB-△F2, and SRB-XhoI-△R2 (Table S1). Next, the mutant protein (*Pc*SRB^Δ130-180^) was expressed and purified using the above experimental method (2.5.). The molecule interaction of *Pc*SRB^Δ130-180^ with WSSV envelope proteins was assessed using the above methods (2.10., 2.12., and 2.13.).

### Phagocytosis rate and phagocytosis index test in hemocytes

Purified WSSV was labeled with fluorescein isothiocyanate (FITC, Beyotime, China, ST2065). The purified WSSV was incubated with FITC for 2 h at 25°C. The sample was washed using PBS four times. The experimental group was injected with labeled WSSV at 24 h after dsRNA injection. The control group was administered PBS. The hemocytes were collected using an anticoagulant (0.14 M NaCl, 0.1 M glucose, 30 mM trisodium citrate, 26 mM citric acid, and 10 mM ethylene diamine tetra acetic acid, pH 4.6) with 4% paraformaldehyde at 1 h after FITC-WSSV injection. Hemocytes were washed three times using PBS. Next, the cell suspension was dropped onto a polylysine-coated glass slide. The samples were incubated with DAPI for 5 min. Samples were observed under a fluorescence microscope (D201, Andor, UK). The phagocytized WSSV and cells were counted in three fields of view. The 10^4^ hemocytes were counted in this assay. The phagocytic rate and index were calculated as 2.9 section. To confirm the result, flow cytometry was also performed. The hemocytes were collected as above in the dark. The cell suspension was filtered using a mesh sieve (diameter: 100 µm; NEST, China). The filtered samples were detected using a flow cytometry analyzer (LSR Fortessa, BD, USA). The hemocytes phagocytizing labeled WSSV were detected. DsGFP injection group was as the mock. The phagocytosis rate was also calculated.

### Lysosome assay

Crayfish for the experiment were randomly selected and divided into five groups, each containing 20 crayfish. PBS was injected into the crayfish in the control group. Chloroquine (CLQ, Selleck, USA, 54057), as a lysosome inhibitor, was injected into the experimental groups at 20, 50, 100, and 200 µg. The number of dead crayfish was recorded. After 1 h of CLQ injection, WSSV was injected into the crayfish. Gills were collected at 24 h after WSSV injection. The mRNA level of *VP28* and WSSV copy number in gills were detected. PBS injection group was used as the mock. The hemocytes were also collected at 24 h after WSSV challenge following dsRNA injection. To analyze the lysosome function, mRNA levels of *LAMP* (XM_04576279) and *CTSL* (XM_045737850) were detected. The lysosomes were labeled using Lyso-Tracker Green (1:2,000, Beyotime, China, C1047S) as the instruction. The hemocytes were collected after *Pc*SRB interference and WSSV challenge as the above. The hemocytes were observed under a confocal microscope (TCS SP8, Leica, Germany). The dsGFP injection group was as the mock. Three crayfish were used for gene expression analyses (*VP28*, *LAMP*, and *CTSL*), and WSSV copy number determination.

### Statistical analysis

Student’s *t*-test was used in the mRNA levels, virus copies assay, and phagocytic rate/index statistic. Significant difference was accepted with 0.01 < *P* < 0.05. Extremely significant difference was accepted with *P* < 0.01. Log-rank (Mantel–Cox) test was used in the survival rate assay. Paired *t*-test was used in the ELISA binding assay.

## RESULTS

### Characterization of *PcSRB* and phylogenetic tree

The full-length cDNA of *PcSRB* was 2,577 bp, and its open reading frame (ORF) region was 1,518 bp, which encoded 505 amino acids (PQ650576, Fig. S1A). The molecular weight of *Pc*SRB protein is approximately 57 kDa, and its isoelectric point is 8.62. *Pc*SRB has a typical B-type scavenger receptor structure that includes cytoplasmic domains at both ends, two transmembrane domains, and a large extracellular domain (Fig. S1C). The SRB sequences from different species were used to construct a phylogenetic tree. SRBs are divided into three branches: Mammalia, Teleosts, and Crustacea. *Pc*SRB was relatively similar to *P. japonicus* and *P. vannamei* (Fig. S1B).

### *Pc*SRB expression

**Fig 1:**
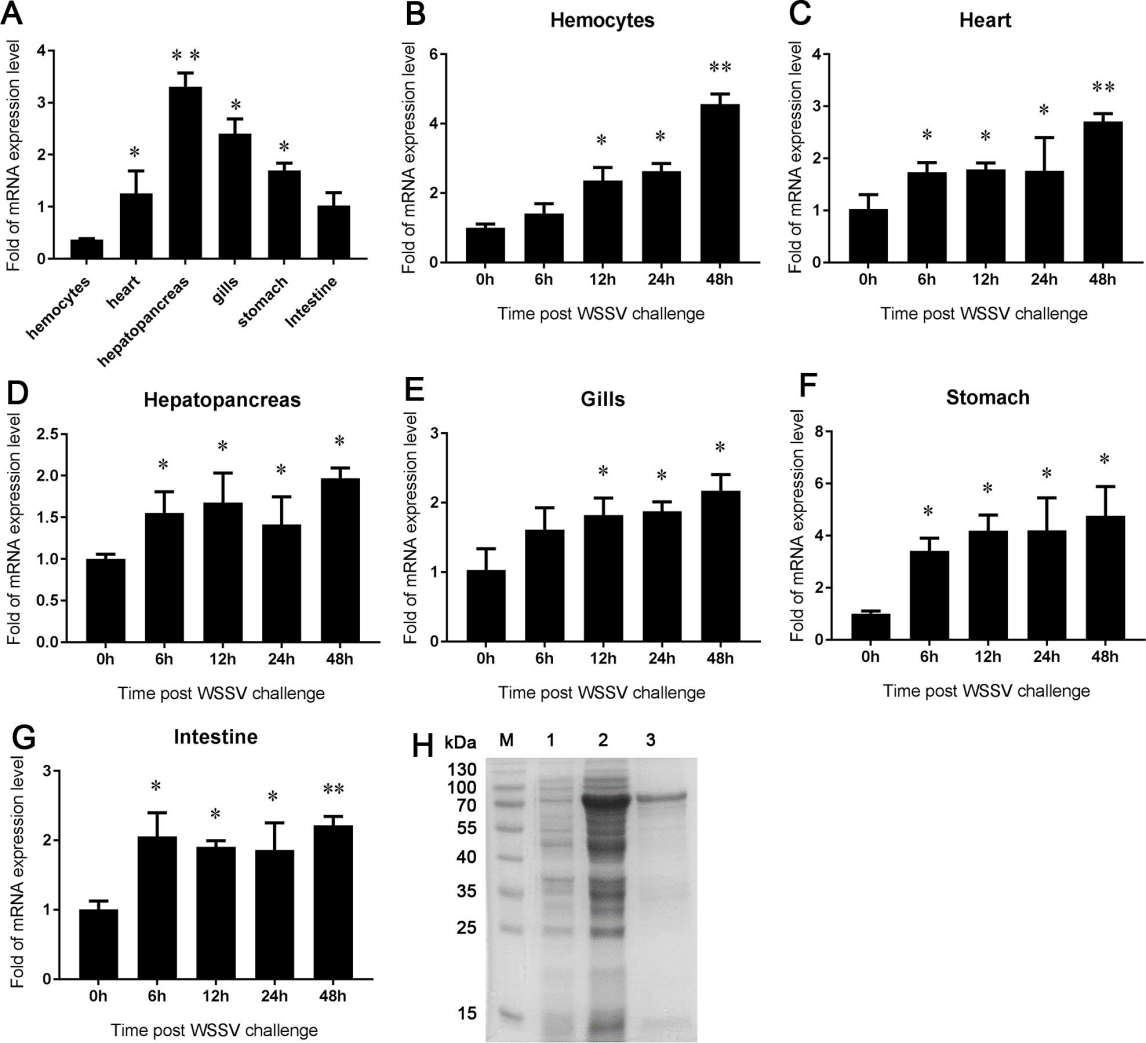
The mRNA levels in different tissues and recombinant expression of *Pc*SRB. (**A**) The mRNA levels of *Pc*SRB in different tissues (hemocytes, heart, hepatopancreas, gills, stomach, and intestine) of non-challenged crayfish. (**B-G**) The *Pc*SRB mRNA levels of *Pc*SRB in tissues after WSSV challenge. Three crayfish were collected at each time point. Each test was performed as three independent repeats. *t*-test was used for significance testing. * 0.01 < *P* < 0.05, ** *P* < 0.01. (**H**) Expression and purification of recombinant *Pc*SRB protein. M, Marker; 1, uninduced sample; 2, induced sample; 3, purified r*Pc*SRB.

We analyzed the tissue distribution in crayfish to study the expression of *Pc*SRB. The results demonstrated that *Pc*SRB was expressed in all the tested crayfish tissues. The level was highest in the hepatopancreas and lowest in hemocytes (Fig. 1A). The expression of *Pc*SRB was upregulated during 6 to 48 h in the heart, hepatopancreas, stomach, and intestine after the WSSV challenge (Fig. 1C, D, F and G). However, the levels were upregulated during 12–48 h in the hemocytes and gills after the virus challenge (Fig. 1B and E). These results indicated that *Pc*SRB was involved in virus-induced immune responses.

### *Pc*SRB affected the survival rate after the WSSV challenge

**Fig 2:**
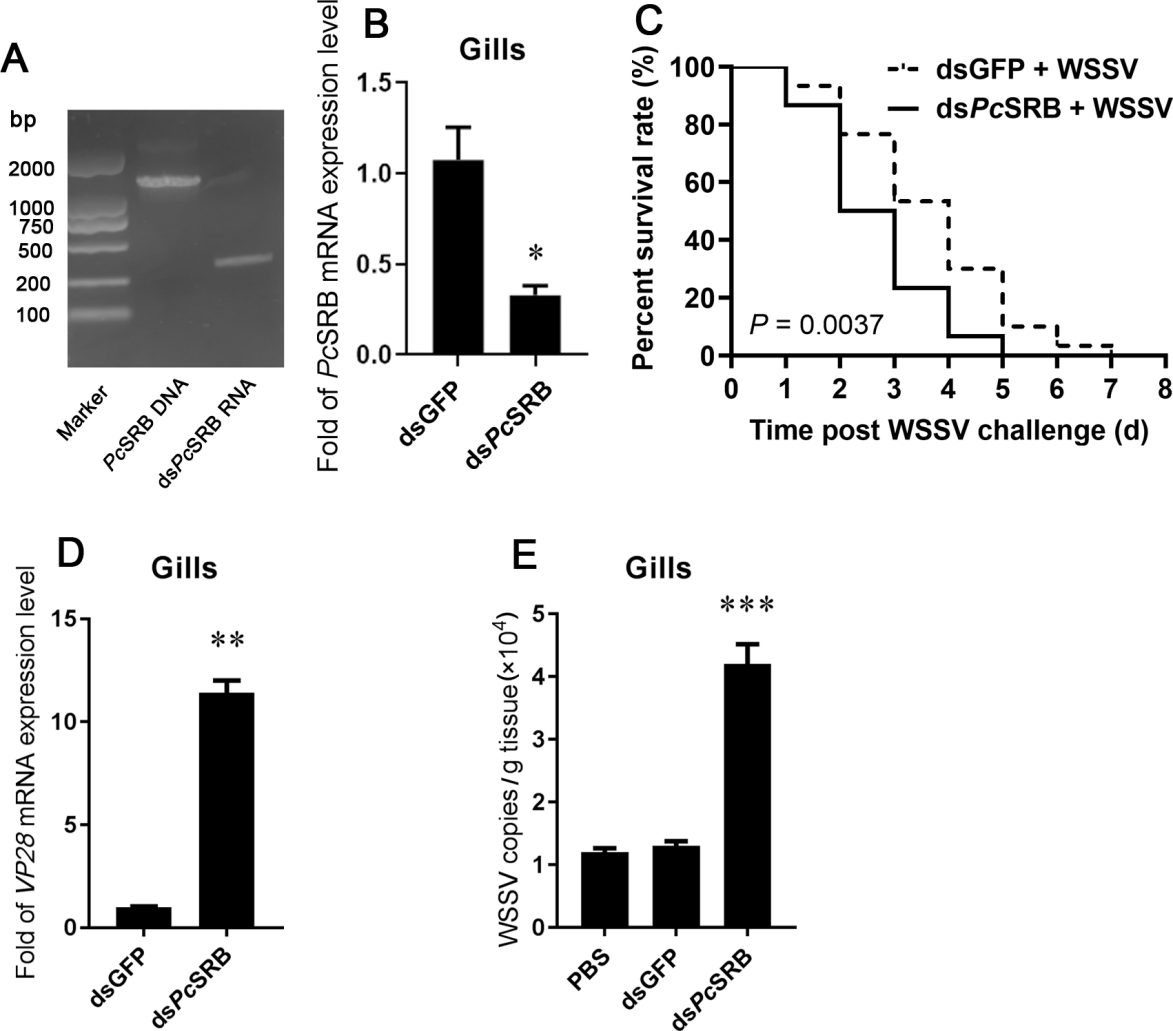
The survival rate and the WSSV copies after *Pc*SRB interference. (**A**) The dsRNA synthesis for *Pc*SRB interference. The lane one presents the full-length *Pc*SRB DNA. Lane two presents the synthesized ds*Pc*SRB RNA. (**B**) *Pc*SRB RNA level detection after dsRNA injection in the gills. (**C**) The survival rate of crayfish after *Pc*SRB interference and WSSV challenge. Thirty crayfish were used in this assay. The assay used Log-rank (Mantel-Cox) Test. (**D**) The relative expression of *VP28* in the gills after *Pc*SRB interference and WSSV challenge. (**E**) The WSSV copies in the gills after *Pc*SRB interference and WSSV challenge. Three crayfish were used in (**B**), (**D**), and (**E**) respectively and each test was repeated thrice. The results were analyzed statistically using a Student’s *t*-test. * 0.01 *< P* < 0.05, ** *P* < 0.01.

Next, we confirmed the influence of *Pc*SRB on the host during the WSSV challenge by conducting RNA interference and survival rate experiments. The results demonstrated that the survival rate of crayfish after ds*Pc*SRB injection was significantly lower than that in the mock group (Fig. 2C). This indicated that *Pc*SRB protected the host during WSSV invasion. In addition, *VP28* gene expression and viral copy numbers were determined. The results demonstrated that *VP28* mRNA amount and the copy number of WSSV after *Pc*SRB interference were greater than that in the mock groups (Fig. 2D and E). These results demonstrated that *Pc*SRB suppressed WSSV replication.

### *Pc*SRB interacted with the WSSV particles

**Fig 3:**
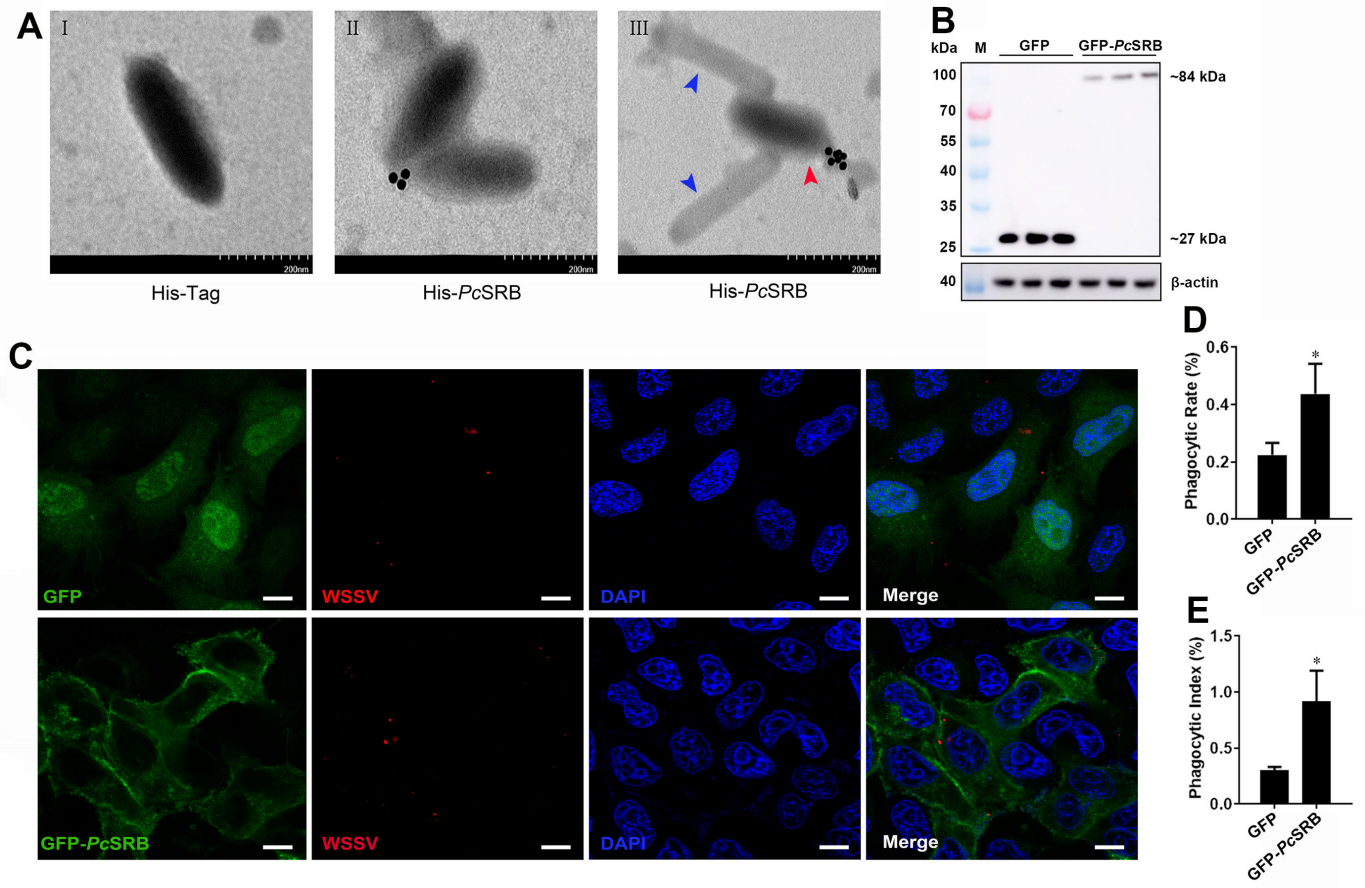
TEM assay and fluorescence detection in cells. (**A**) The interaction detection for *Pc*SRB with WSSV using TEM. The recombinant *Pc*SRB was labeled with colloidal gold particles. The red arrowhead indicates intact WSSV. The blue arrowheads indicate WSSV nucleocapsids. His-*Pc*SRB represents the recombinant protein containing His-Tag. His-Tag group was used as the mock. Scale bar = 200 nm. (**B**) WB detection of *Pc*SRB in HeLa cells. Three independent repeats were performed. (**C**) The co-location and phagocytosis detection of *Pc*SRB with WSSV in HeLa cells. The recombinant plasmid pEGFP-N1-*Pc*SRB was transfected into HeLa cells. The plasmid pEGFP was used as the mock. Blue displays the nucleus. Red displays WSSV, and green displays the GFP or GFP-*Pc*SRB. Scale bar = 10 µm. (**D**) The phagocytic rate of HeLa cells. (**E**) The phagocytic index.

The recombinant *Pc*SRB protein was expressed and purified (Fig. 1H). TEM assay was performed to examine the interaction between *Pc*SRB and WSSV. The results revealed that colloidal gold-labeled *Pc*SRB interacted with WSSV (Fig. 3AII and III). However, the colloidal gold-labeled His-Tag could not bind to WSSV (Fig. 3AI). Moreover, colloidal gold-labeled *Pc*SRB could not interact with WSSV nucleocapsid (Fig. 3AIII). These results indicated that *Pc*SRB can interact with the WSSV envelope. To confirm this hypothesis, a binding assay was performed using HeLa cells. GFP-*Pc*SRB protein was expressed in Hela cells (Fig. 3B). The *Pc*SRB protein was expressed on the cell surface and WSSV particles were binding or phagocytized into HeLa cells. However, WSSV did not bind to the HeLa cells expressing only GFP (Fig. 3C). The phagocytosis rate and the phagocytosis index of cells expressing *Pc*SRB were higher than the mock (Fig. 3D and E). These results suggested that *Pc*SRB interacted with WSSV.

### *Pc*SRB recognized structural proteins of WSSV

**Fig 4:**
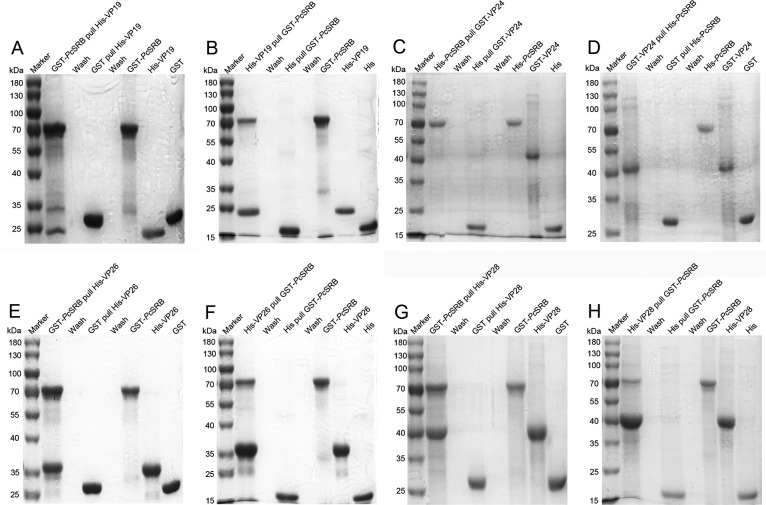
Pulldown assay of *Pc*SRB and WSSV envelope proteins. (**A**) *Pc*SRB pulled virus envelope protein VP19. (**B**) Virus envelope protein VP19 pulled *Pc*SRB. (**C**) *Pc*SRB pulled VP24. (**D**) VP24 pulled *Pc*SRB. (**E**) *Pc*SRB pulled VP26. (**F**) VP26 pulled *Pc*SRB. (**G**) *Pc*SRB pulled VP28. (**H**) VP28 pulled *Pc*SRB. “Wash” lanes present the last wash eluate samples.

Pull-down experiments were performed to study the interacted pattern of *Pc*SRB and WSSV. The results demonstrated that *Pc*SRB bound to envelope proteins VP19, VP26, and VP28 (Fig. 4A, B, E, F, G, and H). However, *Pc*SRB did not bind to VP24 (Fig. 4C and D). These results indicated that *Pc*SRB recognizes specific envelope proteins.

### *Pc*SRB recognized lipid molecule of WSSV

A previous study reported that if a protein interacted with small molecules, the protein absorbance of UV–visual spectroscopy would change (31). To determine whether *Pc*SRB interacts with the lipid molecules of WSSV, a spectroscopic assay was performed. The results demonstrated that the absorbance ranks of *Pc*SRB and CD3,5 were wider than those of the mock group (Fig. S2A and B). However, the absorbance rankings of *Pc*SRB and DBP were not significantly different from those of the mock group (Fig. S2C and D). These results indicated that the *Pc*SRB protein could interact with CD3,5 lipids rather than with DBP.

**Fig 5:**
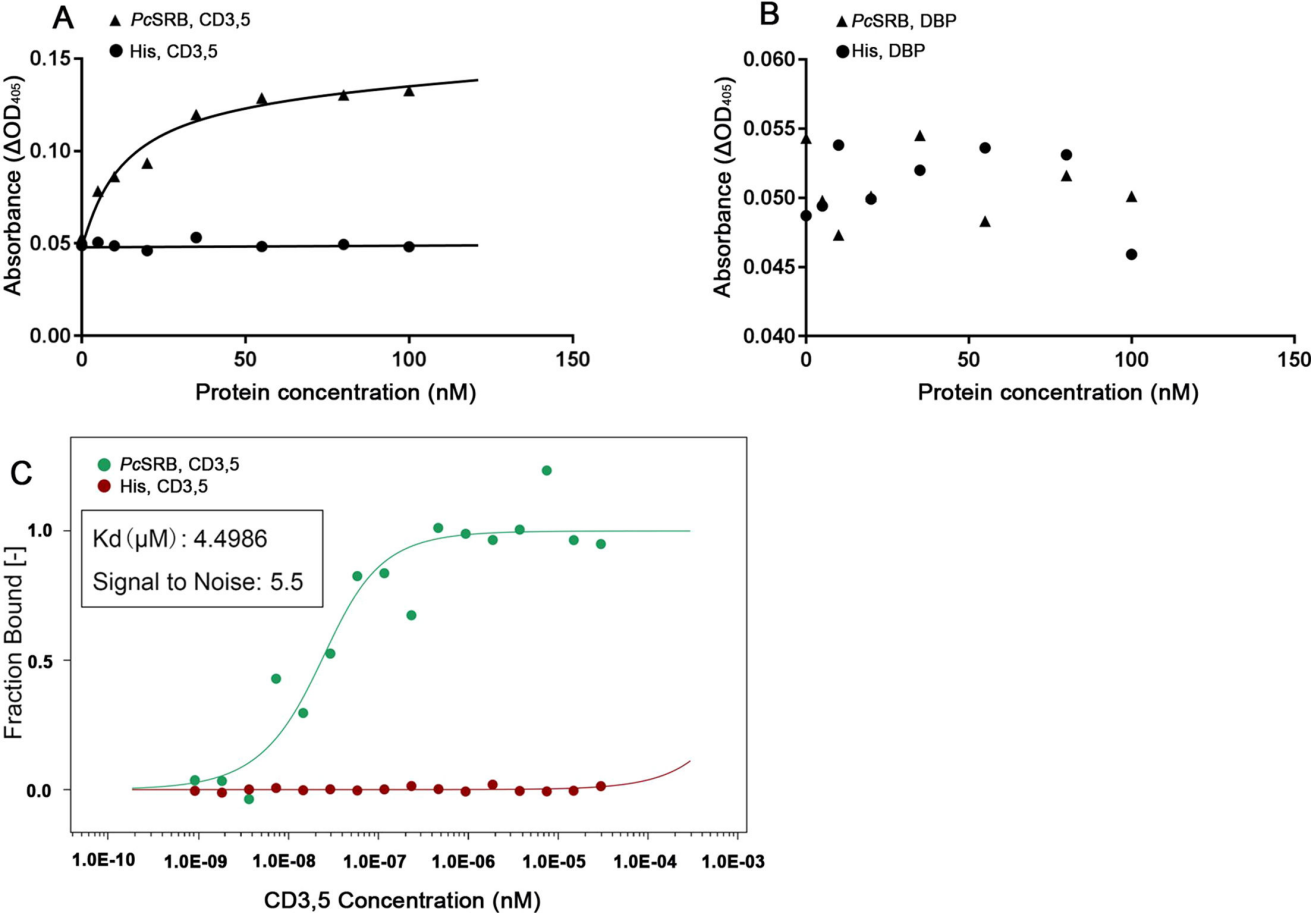
Combination assay for *Pc*SRB and lipid molecules. (**A**) The ELISA assay for *Pc*SRB and CD3,5. His-Tag was the mock group. (**B**) The ELISA assay for *Pc*SRB and DBP. His-Tag was the mock group. (**C**) MST assay for *Pc*SRB and CD3,5. Kd, equilibrium dissociation constant, represents the affinity of *Pc*SRB and CD3,5. Signal to noise represents experimental credibility (signal to noise >5).

Next, we performed ELISA to investigate the binding of *Pc*SRB to lipid molecules. The results demonstrated that *Pc*SRB could bind to CD3,5 rather than DBP (Fig. 5A and B). MST experiments were performed to confirm this hypothesis. A binding curve could be observed with a Kd value at 4.4986 µM. However, the His-Tag sample did not form a binding curve (Fig. 5C). These results suggested that *Pc*SRB interacted with CD3,5. In other words, *Pc*SRB specifically recognized the lipid molecules of WSSV.

### Hydrophobic domain of *Pc*SRB affected the combination of VP proteins and lipid molecule

**Fig 6:**
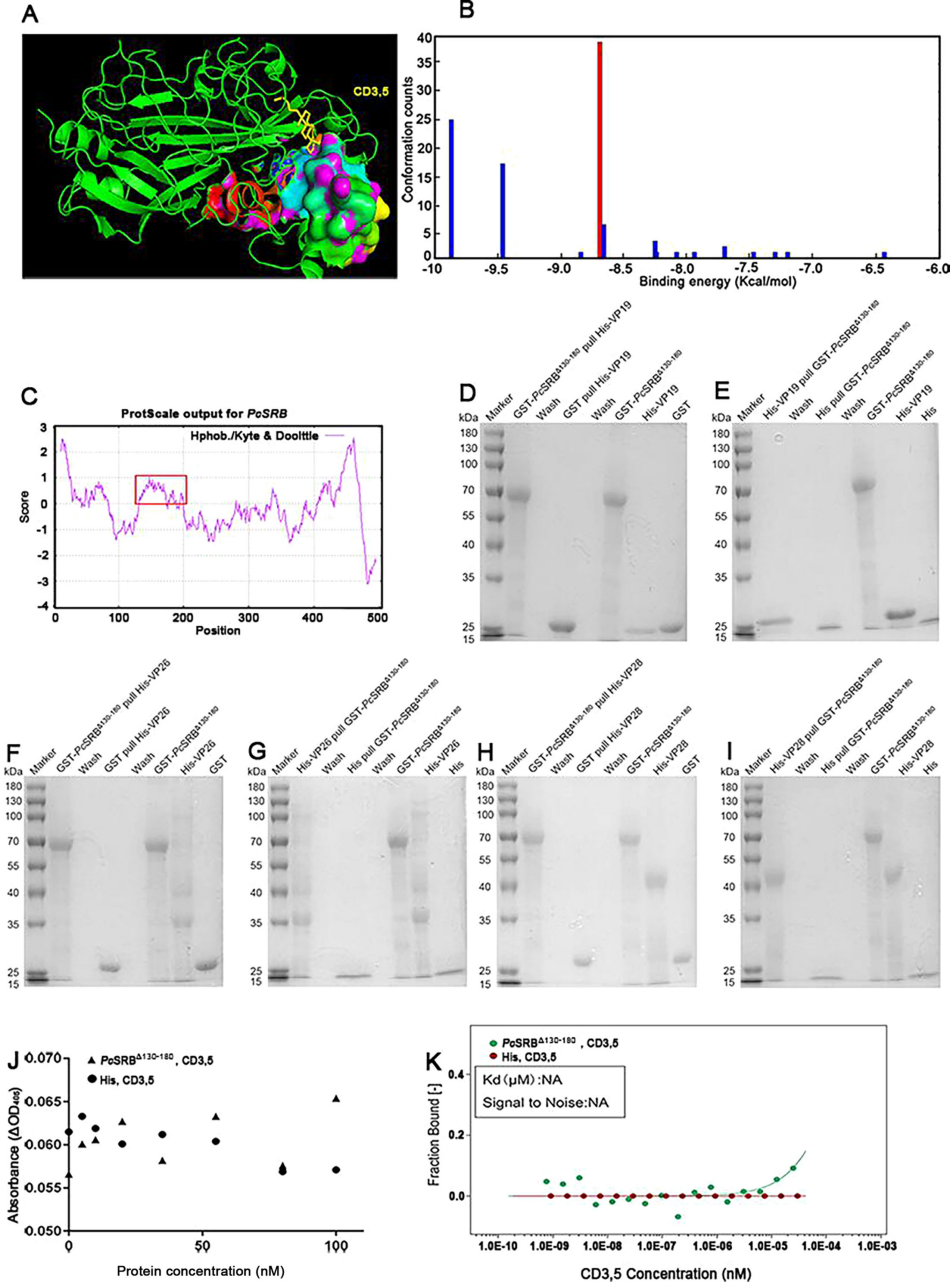
Mutant *Pc*SRB^Δ130-180^ building and the interaction assay. (**A**) Prediction model for *Pc*SRB binding to CD3,5. CD3,5 is yellow labeled. The interaction structure is depicted as a surface image. (**B**) The cluster analysis of predicted models for binding. One hundred times were performed in the calculation. The most credible pattern is presented in the red column. (**C**) *Pc*SRB hydrophobicity assay. (**D**) *Pc*SRB^Δ130-180^ pulled virus envelope protein VP19. (**E**) VP19 pulled *Pc*SRB^Δ130-180^. (**F**) *Pc*SRB^Δ130-180^ pulled VP26. (**G**) VP26 pulled *Pc*SRB^Δ130-180^. (**H**) *Pc*SRB^Δ130-180^ pulled VP28. (**I**) VP28 pulled *Pc*SRB^Δ130-180^. (**J**) The ELISA assay for *Pc*SRB^Δ130-180^ and CD3,5. His-Tag was the mock group. (**K**) MST assay diagram for *Pc*SRB^Δ130-180^ and CD3,5. NA: not available.

We next identified the key domain for recognition by scanning the potential binding positions for the whole *Pc*SRB molecule. The binding model was constructed using Autodock software. A cluster analysis of the predicted models is shown (Fig. 6B). The most credible model pattern is depicted in red. The potential binding pattern was 39 over 100 times fell in that pattern. The binding energy was −8.69. A hydrophobicity assay was performed to analyze the characteristics of *Pc*SRB. The predicted binding region overlapped with the region from 130 to 180 of the amino acid sequence presented using a red rectangle, which showed highly hydrophobic characteristics, except for the transmembrane domains (Fig. 6C). This indicated that the interaction position was perhaps involving the *Pc*SRB hydrophobic domain. The hydrophobic domain deletion mutant protein (*Pc*SRB^Δ130-180^) was expressed (Fig. S3). Pull-down, ELISA, and MST experiments were performed. The results showed that *Pc*SRB^Δ130-180^ could not interact with the WSSV structure proteins, VP19, VP26, and VP28 (Fig. 6D through I). In addition, *Pc*SRB^Δ130-180^ could not interact with the lipid molecule CD3,5 (Fig. 6J and K). These results indicated that the hydrophobic domain is essential for recognizing WSSV.

### *Pc*SRB promoted hemocyte phagocytosis

**Fig 7:**
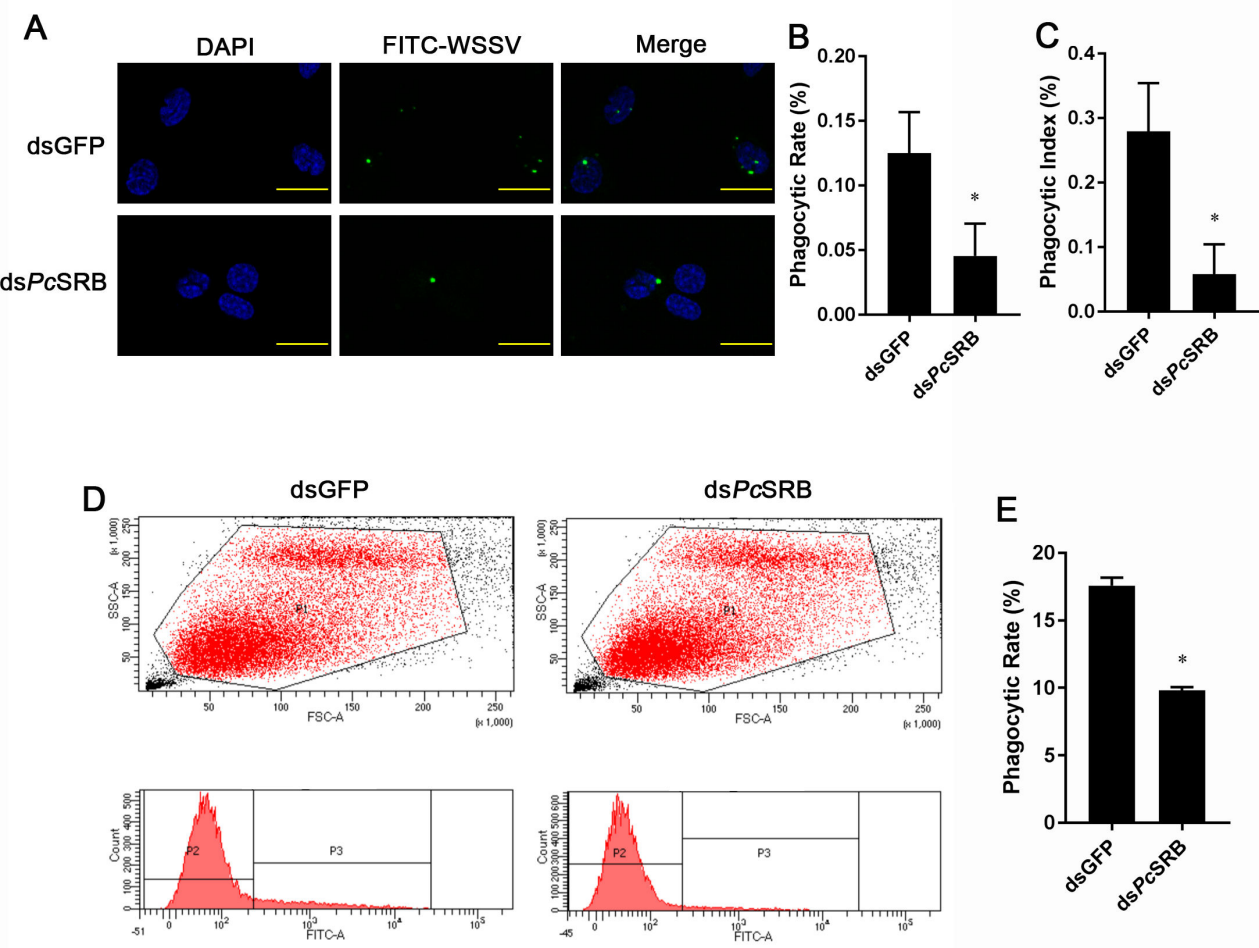
Phagocytosis assay of hemocytes. (**A**) Phagocytosis of WSSV by hemocytes. The hemocytes were collected at 1 h after WSSV challenge following ds*Pc*SRB injection. WSSV virus was labeled by FITC. The nuclei were stained with DAPI. The number of WSSV was counted using a fluorescence microscope. Scale bar = 20 µm. (**B**) The phagocytic rate of hemocytes. (**C**) The phagocytic index. (**D**) Flow cytometry detection of phagocytosis of WSSV in the hemocytes. P1 represents the intact hemocyte population. P3 represents the intact hemocyte containing FITC-WSSV. (**E**) The phagocytosis rate was detected using flow cytometry. The results were analyzed statistically using a Student’s *t*-test. * 0.01 *< P* < 0.05.

The above studies have demonstrated that *Pc*SRB can recognize WSSV and inhibit viral proliferation in crayfish. To study the mechanism of viral resistance, we studied the phagocytosis of WSSV particles in hemocytes after *Pc*SRB interference. The results demonstrated that the phagocytic rate and phagocytic index of hemocytes in the interference group were significantly lower than those in the mock group (Fig. 7A through C). To confirm these results, we performed flow cytometry to detect the phagocytosis of hemocytes. The results demonstrated that the hemocytes containing labeled WSSV were significantly fewer in the interference group (Fig. 7D and E). These results showed that *Pc*SRB could promote the phagocytosis of WSSV in crayfish hemocytes.

### Lysosomes participate in the clearance of WSSV

**Fig 8:**
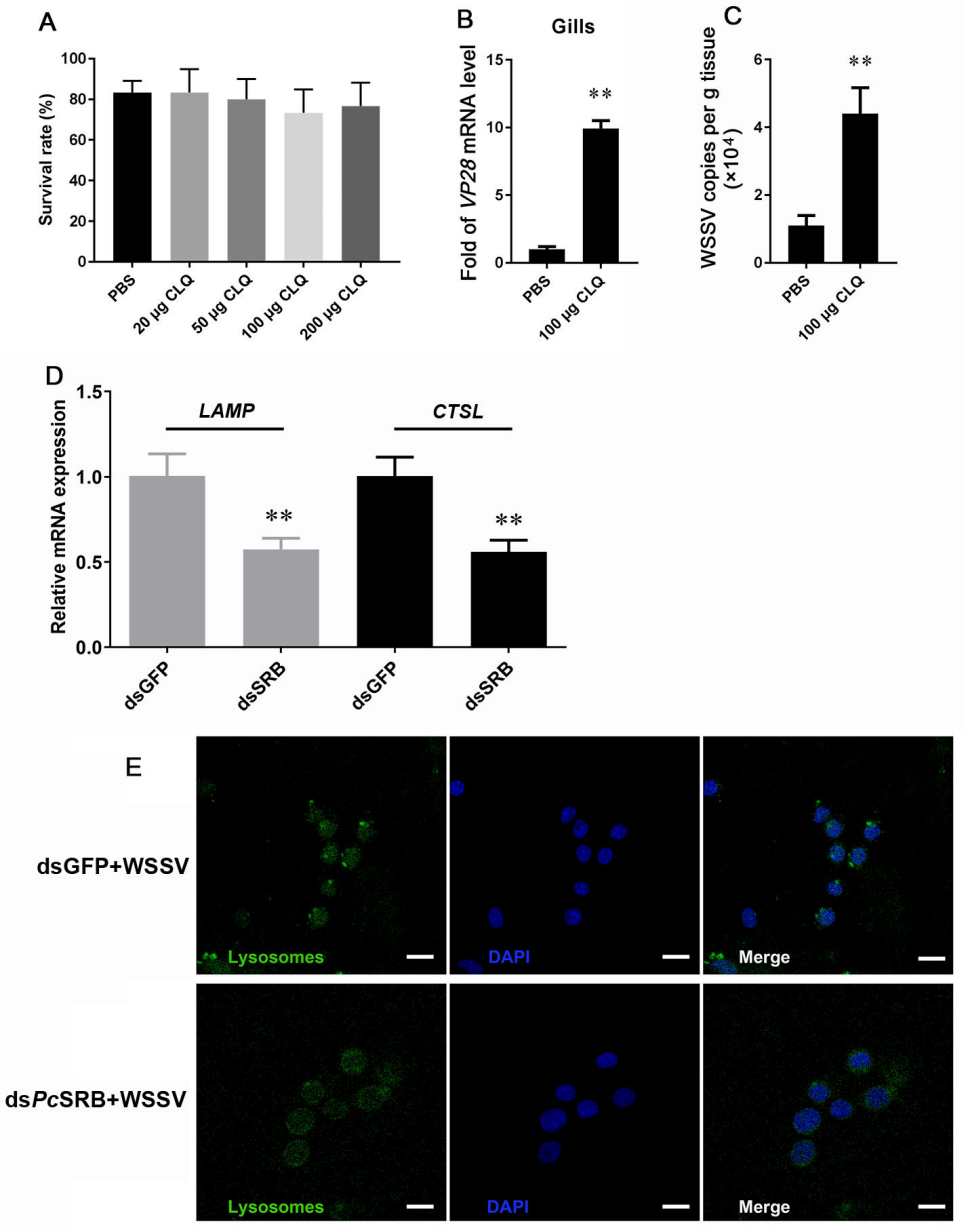
WSSV and lysosome detection. (**A**) The CLQ effect on the survival rate of crayfish. One hundred crayfish were used in this test. These crayfish were divided equally into five groups. CLQ (20, 50, 100, and 200 µg) was injected respectively in these groups. (**B**) *VP28* expression in the gills. (**C**) WSSV copies in crayfish. The mRNA level and WSSV copies were detected at 24 h after WSSV injection following CLQ injection. (**D**) *LAMP* and *CTSL* mRNA levels in hemocytes. The hemocytes were collected at 24 h after WSSV injection following dsRNA injection. Each test was repeated thrice in (**B**), (**C**), and (**D**). The results were analyzed statistically using a Student’s *t*-test. ** *P* < 0.01. (**E**) Lysosome detection in hemocytes. The hemocyte collection method was the same as (**D**). The lysosomes were displayed using Lyso-Tracker Green reagent. The nuclei were stained with DAPI. Scale bar = 10 µm.

Next, we analyzed lysosomal function to study how hemocytes eliminated WSSV. CLQ was used to inhibit lysosomal activation. The results demonstrated that low-dose CLQ stimulation did not have a significant effect on crayfish survival (Fig. 8A). In addition, the expression of *VP28* in the gills was significantly upregulated in the lysosome-inhibited group (Fig. 8B). Simultaneously, the number of WSSV copies in the crayfish was higher in the CLQ group than in the mock group (Fig. 8C). These results suggested that lysosome could inhibit the replication of WSSV. The function of lysosomes was due to the component protein and lysosomal protease (32). In this study, one vital component protein gene *LAMP* and an important lysosomal protease gene *CTSL* were detected. The mRNA levels of *LAMP* and *CTSL* were lower after WSSV challenge in the *Pc*SRB knockdown group than the mock (Fig. 8D). The lysosomes were also displayed. The number of lysosomes was fewer in *Pc*SRB knockdown group than the mock. Additionally, the luminance was weaker in *Pc*SRB knockdown group than the mock (Fig. 8E). These results suggested that *Pc*SRB could influence lysosomal structure and function and promote WSSV elimination.

## DISCUSSION

In this study, we cloned the class B scavenger receptor from *P. clarkii* and termed it *Pc*SRB. The expression of *Pc*SRB significantly increased after the WSSV challenge. *Pc*SRB suppresses WSSV replication. *Pc*SRB could interact with both the lipid molecule CD3,5 and the VPs of WSSV. The hydrophobic domain is also important for recognition. Furthermore, *Pc*SRB inhibited WSSV replication by promoting lysosomal activity. These results indicate that *Pc*SRB plays a vital role in WSSV infection.

WSSV is a lethal crustacean pathogen. It is important to understand the defense mechanism by which the host recognizes the virus during pathogen invasion. *Mj*SRC from kuruma shrimp recognizes WSSV by interacting with VP19 (17). At the same time, *Mj*SRC promotes bacterial clearance by enhancing hemocyte phagocytosis and AMP expression (33). These facts indicate that SRs could be involved in both the WSSV infection and bacterial invasion. Previous studies have demonstrated that SRBs recognize bacteria and promote AMP expression (34–36). In this study, we found that *Pc*SRB was highly expressed after the WSSV challenge and restricted viral replication (Fig. 1 and 2). In addition, *Pc*SRB interacted with VP19, VP26, and VP28 rather than with VP24 (Fig. 4). These results suggest that SRB could participate in the defense against both bacterial invasion and WSSV infection.

WSSV is a virus packaged in an envelope. Therefore, the VPs located in the envelope interact with several receptors (37). The lipids of the envelope are considered to be derived from the host membrane. Therefore, these lipids have not been studied as pathogen-associated molecular patterns (PAMPs) (38). In recent years, WSSV lipids have been analyzed, and four different lipid molecules have been identified (22). In this study, we determined whether *Pc*SRB interacts with CD3,5 or DBP contained in the envelope. Interestingly, the results demonstrated that *Pc*SRB could interact with CD3,5 rather than DBP (Fig. S2; Fig. 5). These results indicate that lipids located in the envelope could be novel ligands for host receptors and trigger immune responses.

SRs are important for pathogen recognition. SRB1 in human hepatic cells acts as a cell surface-binding receptor for the dengue virus NS1 (39). SRB1 can bind to large (L) surface proteins of the hepatitis B virus (40). A subsequent study found Pre-S1 domain of the L protein is the binding region for SRB1 (41). The scavenger receptor cysteine-rich domain of pig CD163 participates in porcine reproductive and respiratory syndrome virus infection (42). SRB1 co-localizes with angiotensin-converting enzyme 2 and promotes severe acute respiratory syndrome coronavirus type 2 internalization (43). In addition, SRBs recognize bacteria by binding to PAMPs. SRBs in crustaceans can interact with lipopolysaccharides and lipoteichoic acid in marine crabs (35, 44). However, the ligand-binding domains of SRBs have not yet been elucidated. In the present study, we predicted the binding domain of *Pc*SRB and constructed a *Pc*SRB mutant. The results demonstrated that the domain of *Pc*SRB from 130–180 amino acid was important for binding to CD3,5, and the VPs of WSSV (Fig. 6). The binding domain of *Pc*SRB forms a hydrophobic pocket for lipid molecule and VPs interaction. These results indicated that the hydrophobic region of *Pc*SRB is vital for recognizing WSSV.

Lysosomes participate in the internalization and clearance of several viruses. Membrane-associated RING-CH proteins can activate lysosomes to degrade influenza A viruses (45). Heat shock protein family A member 5 promotes viral trafficking via the endo/lysosomal pathway (46). Autophagy receptors could bind to envelope proteins of HBV and trigger the Rab9-dependent lysosomal degradation pathway to degrade the virus (47, 48). Cathepsin proteases are important for lysosomal acidity and degradation. However, LAMP is associated with lyososomal exocytosis. Additionally, LAMP1 is the most abundant lysosomal membrane protein accounting for 50% of this membrane’s total protein (49). These studies demonstrate that lysosomes play a vital role in viral clearance. In this study, we found that *Pc*SRB promoted the phagocytosis of hemocytes by WSSV (Fig. 7). However, lysosome activity inhibition facilitated WSSV replication (Fig. 8C). This is consistent with the consequence after *Pc*SRB interference (Fig. 2E). Therefore, whether the receptor *Pc*SRB affects lysosome activity is meaningful for the study. The results indicated that *Pc*SRB could promote lysosomes by affecting the lysosome quantity and expression of lysosomal protein (CTSL and LAMP) (Fig. 8D and E). Therefore, *Pc*SRB could promote lysosome activity and then inhibit WSSV replication in hemocytes. However, how *Pc*SRB affects the lysosome should be uncovered in further study.

**Fig 9:**
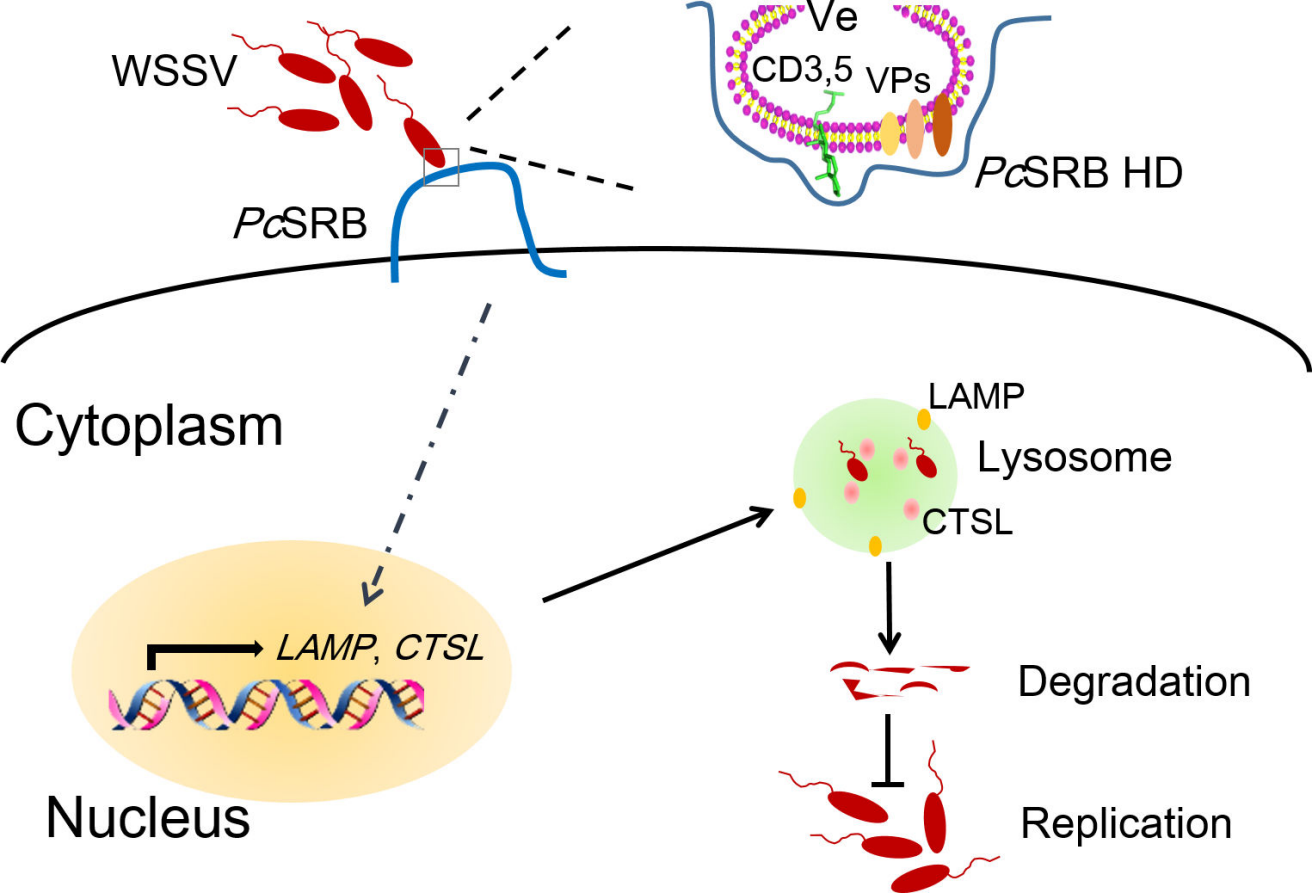
Graphic illustration of the conclusions. Ve, WSSV envelope. *Pc*SRB HD, *Pc*SRB hydrophobic domain (130–180 aa).

In conclusion, we identified a receptor homologous to the SRBs of vertebrates, designated *Pc*SRB. *Pc*SRB can recognize WSSV binding to envelope lipid molecules CD3,5 and VPs. This recognition is dependent on *Pc*SRB hydrophobic region (130–180 aa). *Pc*SRB promotes the lysosome activity to accelerate WSSV phagocytosis and inhibits virus replication (Fig. 9). This study provides a novel virus recognition mechanism of SRBs different from interacting with viral protein. Our study also exhibits a viral defense mechanism for *Pc*SRB inhibiting WSSV via the lysosome pathway.

## Data Availability

The genome sequence of *PcSRB* has been deposited to NCBI GenBank under accession number PQ650576.

## SUPPLEMENTAL MATERIAL

Supplemental material jvi.01700-24-s0001.docx Figures S1 to S4; Table S1.

ASM does not own the copyrights to Supplemental Material that may be linked to, or accessed through, an article. The authors have granted ASM a non-exclusive, world-wide license to publish the Supplemental Material files. Please contact the corresponding author directly for reuse.
