# Supplementary material for: Class B scavenger receptor resists WSSV replication by recognizing the viral lipid molecule and promoting phagocytosis
Source: J Virol. 2025 Feb 5;99(3):e01700-24. doi: 10.1128/jvi.01700-24 (PMC11915803; doi:10.1128/jvi.01700-24)
Supplement: Supplemental material — Figures S1 to S4; Table S1. [file jvi.01700-24-s0001.docx]

**Supplemental Figures**


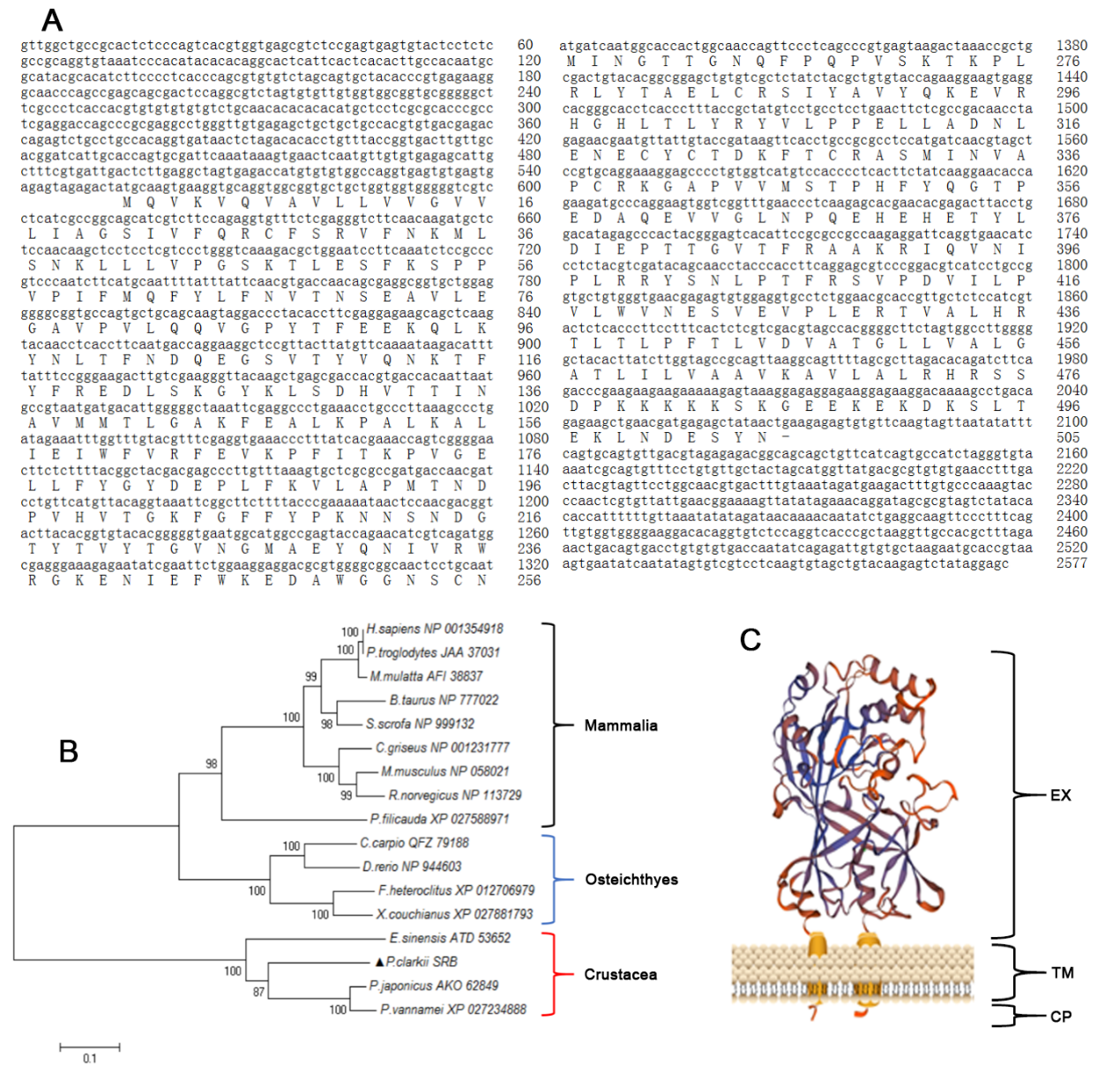


Figure S1.


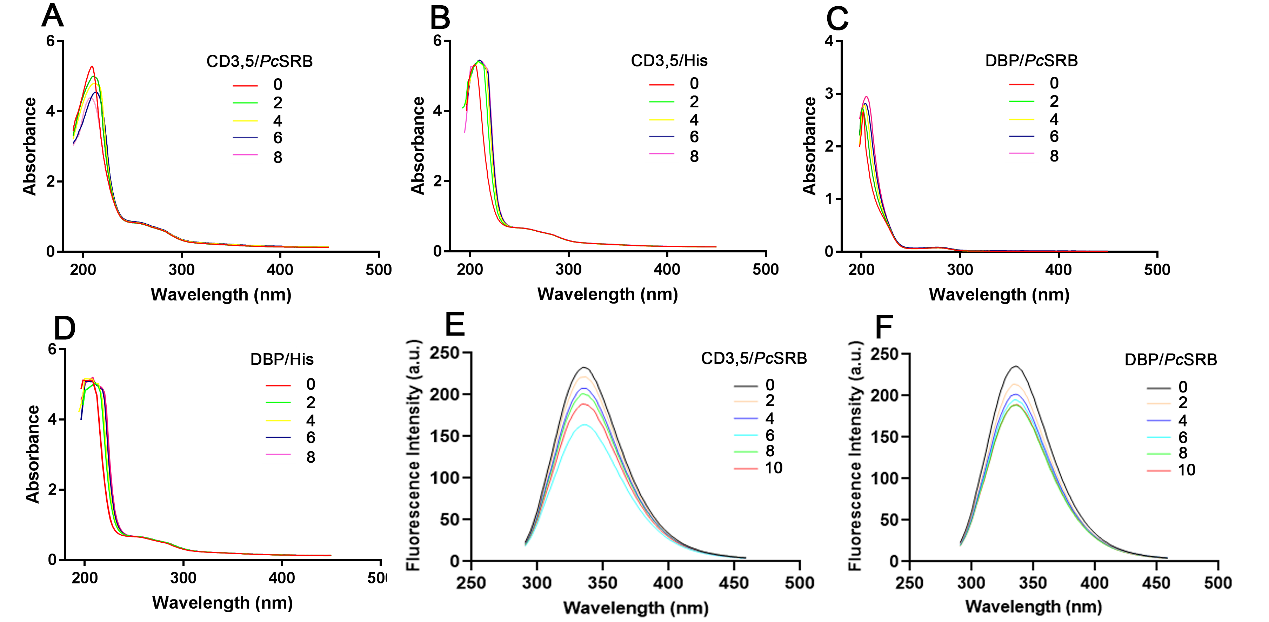


Figure S2.


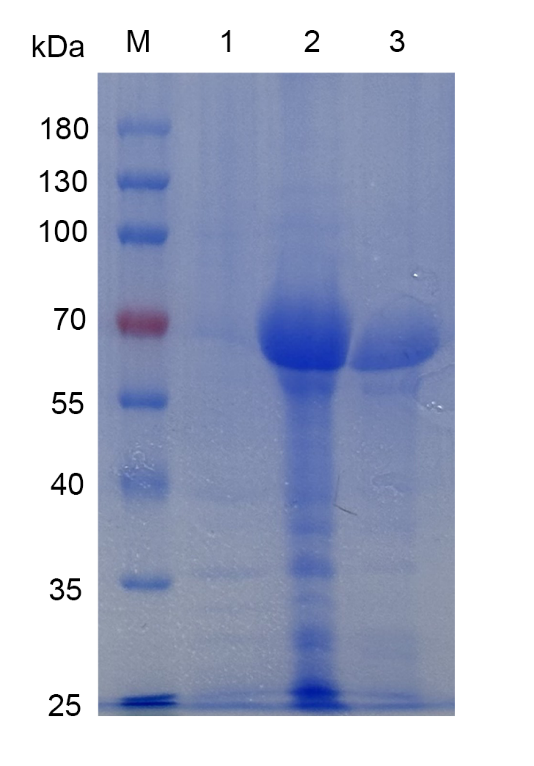


Figure S3.

**
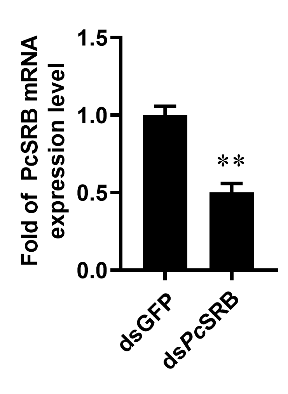
**

Figure S4

**Supplemental Legends**

**Figure S1. The bioinformatics of *Pc*SRB.** (A) The nucleotide and amino acid sequences of *Dr*RBL. The translation was performed using the online ExPASy-Translate tool. (B) Evolutionary tree analysis of SRBs. The distance calculation was performed using the neighbor-joining (NJ) method. Branch confidence levels are built on 1,000 bootstrap replicates. The SRBs sequences used in this study were from different species, *Homo sapiens*, *P. troglodytes*, *Macaca mulatta*, *Bos taurus*, *Sus scrofa*, *Cricetulus griseus*, *Mus musculus*, *Rattus norvegicus*, *Cyprinus carpio*, *Danio rerio*, *Fundulus. heteroclitus*, *Xiphophorus couchianus*, *Eriocheir sinensis*, *Penaeus. japonicus*, *Penaeus vannamei*. (C) The three-dimensional model of *Pc*SRB. The model was built using SWISS-MODEL (<https://swissmodel.expasy.org/>). EX, the extracellular domain; TM, the transmembrane domain; and CP, the intracellular domain.

**Figure S2. UV-Vis spectroscopy and fluorescence spectroscopy of *Pc*SRB and lipid molecules.** (A)–(D) UV–Vis spectroscopy. His groups were as the mocks. (E and F) Fluorescence spectroscopy. The concentration ratio of lipid molecules (CD3,5, DBP) and *Pc*SRB was as follows: 0, 2, 4, 6, 8, 10.

**Figure S3.** Expression and purification of mutant *Pc*SRB^Δ130-180^. M, Marker; 1, uninduced sample; 2, induced sample; 3, purified *Pc*SRB^Δ130-180^.

**Figure S4.** *Pc*SRB RNA level detection after dsRNA injection in the hemocytes. Three crayfish were used. Each test was repeated thrice. The results were analyzed statistically using a Student’s *t-*test. ** *P* < 0.01.

**Supplemental Table**

**Table S1. Sequences of primers used in this study**

| **Primer name** | **Primer sequence Sequence (5’-3’)** |
| --- | --- |
| SRB-F | ATGCAAGTGAAGGTGCA |
| SRB-R | TCAGTTATAGCTCTCAT |
| SRB-RT-F | TCGCTCTATCTACGCT |
| CTSL-RT-F | TGCCTCACATGGCTCAATCC |
| CTSL-RT-R | AGGAAGGGCAAGAGTTCGG |
| LAMP-RT-F | GTTCGAGACATAAGTCCCCCG |
| LAMP-RT-R | CAATGGTTACGCTTTCCCGC |
| SRB-RT-R | TCAAACCGACCACTT |
| VP28-RT-F | AAAACCTCCGCATTCCT |
| VP28-RT-R | TCCGCATCTTCTTCCTT |
| 18S-RT-F | TCTTCTTAGAGGGATTAGCGG |
| 18S-RT-R | AAGGGGATTGAACGGGTTA |
| GFPi-F | GCGTAATACGACTCACTATAGGTGGTCCCAATTCTCGTG |
| GFPi-R | GCGTAATACGACTCACTATAGGCTTGAAGTTGACCTTGA |
| SRBi-F | GCGTAATACGACTCACTATAGGGCTGGAATCCTTCAAAT |
| SRBi-R | GCGTAATACGACTCACTATAGGCTCGTCGTAGCCGTAAA |
| SRB-BamHI-F | CGCGGATCCAGGGTCTTCAACAAGAT |
| SRB-XhoI-R | CCGCTCGAGTCAGGCTACGTCGACGAGAGT |
| VP19-BamHI-F | CGCGGATCCATGGCCACCACGACTAACA |
| VP19-XhoI-R | CCGCTCGAGTTACTGCCTCCTCTTGG |
| VP24-BamHI-F | CGCGGATCCATGCACATGTGGGGGGTTTA |
| VP24-XhoI-R | CCGCTCGAGTTATTTTTCCCCAACCTT |
| VP26-BamHI-F | CGCGGATCCATGGAATTTGGCAACCTAA |
| VP26-HindIII-R | CCCAAGCTTTTACTTCTTCTTGATTTCGT |
| VP28-BamHI-F | CGCGGATCCATGGATCTTTCTTTCACTCTTT |
| VP28-XhoI-R | CCGCTCGAGTTACTCGGTCTCAGTGCCA |
| SRB-BamHI-△F1 | CGCGGATCCAGGGTCTTCAACAAGAT |
| SRB-△R1 | CAAGGGCTCGTCGTAGCCGTGGTCGCTCAGCTTGTAA |
| SRB-△F2 | TTACAAGCTGAGCGACCACGGCTACGACGAGCCCTTG |
| SRB-XhoI-△R2 | CCGCTCGAGTCAGGCTACGTCGACGAGAGT |
| eSRB-HindIII-F | CCCAAGCTTGCCACCATGCAAGTGAAGGTGCA |
| eSRB-BamHI-F | CGCGGATCCCGTCAGTTATAGCTCTCAT |
